# Supplementary material for: Novel Black Seed Polysaccharide Extract-g-Poly (Acrylate) pH-Responsive Hydrogel Nanocomposites for Safe Oral Insulin Delivery: Development, In Vitro, In Vivo and Toxicological Evaluation
Source: Pharmaceutics. 2022 Dec 25;15(1):62. doi: 10.3390/pharmaceutics15010062 (PMC9864008; doi:10.3390/pharmaceutics15010062)
Supplement: Supplementary file 1 [file pharmaceutics-15-00062-s001.zip › pharmaceutics-2070702-supplementary.pdf]

pharmaceutics

MDPI

Supplementary Material

Table S1. Correlation coefficient (R^2) and release rates of all Ins-BA hydrogel and Ins-Mmt-BA hydrogel NC formulations

Formulation Codes	Release Model										
	Zero order		First order		Higuchi		Korsemeyer-Peppas			Hixson-Crowell	
	R^2	K_o (h^{-1})	R^2	K_1 (h^{-1})	R^2	k_H (h^{-1})	R^2	k_{KP} (h^{-1})	n	R^2	k_{HC} (h^{-1})
Ins-BA1	0.9565	8.169	0.8629	0.128	0.7643	22.425	0.9752	4.280	1.289	0.8971	0.037
Ins-BA2	0.9493	7.056	0.8607	0.101	0.7367	19.196	0.9897	2.423	1.476	0.8907	0.030
Ins-BA3	0.9413	6.664	0.8568	0.093	0.7243	18.081	0.9889	2.011	1.533	0.8853	0.028
Ins-BA4	0.9558	7.200	0.8681	0.105	0.7475	19.636	0.9901	2.758	1.428	0.8983	0.031
Ins-BA5	0.9601	7.480	0.8713	0.111	0.7570	20.452	0.9883	3.220	1.377	0.9024	0.033
Ins-BA6	0.9613	8.519	0.8647	0.137	0.7746	23.443	0.9761	4.865	1.251	0.9003	0.040
Ins-Mmt-BA1	0.9929	3.955	0.9369	0.063	0.8432	15.455	0.9934	3.544	1.038	0.9628	0.018
Ins-Mmt-BA2	0.9895	3.702	0.9363	0.057	0.8277	14.403	0.9915	2.947	1.078	0.9603	0.017
Ins-Mmt-BA3	0.9857	3.514	0.9326	0.052	0.8128	13.615	0.9900	2.474	1.120	0.9556	0.015
Ins-Mmt-BA4	0.9914	3.776	0.9396	0.059	0.8359	14.723	0.9924	3.212	1.056	0.9636	0.017
Ins-Mmt-BA5	0.9927	3.873	0.9388	0.061	0.8407	15.121	0.9933	3.400	1.045	0.9637	0.018
Ins-Mmt-BA6	0.9936	4.072	0.9386	0.067	0.8527	15.962	0.9936	3.961	1.010	0.9637	0.018
